# Association of hepatitis B infection with high-risk complications in total joint arthroplasty

**DOI:** 10.1186/s12891-019-2535-y

**Published:** 2019-04-11

**Authors:** Jin Wang, Guanglei Zhao, Jie Chen, Jun Xia, Siqun Wang, Gangyong Huang, Feiyan Chen, Jingsheng Shi, Yuanqing Yang, Yibing Wei

**Affiliations:** Division of Orthopaedic Surgery, Huashan Hospital, Fudan University, Shanghai, 200040 China

**Keywords:** Total joint arthroplasty, Hepatitis B infection, Risk factor, Complication

## Abstract

**Background:**

An increasing number of patients with hepatitis B virus (HBV) infection are undergoing total joint arthroplasty (TJA) surgery in China. Less attention is provided to the special populations and the purpose of this study is to assess the effect of HBV infection on the prognosis TJAs.

**Methods:**

We retrospectively reviewed patients who underwent elective primary hip and knee arthroplasties in Shanghai Huashan Hospital from 2013 to 2016. Non-hepatitis B cohort was built to match the case cohort to identify whether HBV infection was a risk factor associated with postoperative complications. A total number of 196 patients who underwent primary TJAs were involved in the study, including 49 patients with hepatitis B and 147 non-hepatitis B subjects.

**Results:**

Among all the patients with TJAs, 5.5% of patients were infected with HBV for the first time. The incidence rate of complications in patients after arthroplasty with hepatitis B infection was significantly higher than that in patients without hepatitis B (10.2% compared to 4.7%, *P* < 0.01). Surgical related complications (6.1% compared to 3.4%) and general medical complications (4.1% compared to 1.3%) were higher than those in non-B hepatitis group. Compared with non-B hepatitis group, the overall risk of hepatitis B infection increased by 25% (95% CI, 1.04–1.46; *p* < 0.01). Similar results were obtained for medical and surgical complications. HBV infection presented a 31% increased risk (95% CI, 1.02–1.62; *p* < 0.01) for medical complication and an 18% increased risk (95% CI, 1.10–1.26; *p* < 0.01) for surgical complication. No statistical difference was found between the surgical methods and sex. However, a significant difference of C-reactive protein (CRP) level was found between HBV infection group and the matched non-infected group (*P* < 0.01).

**Conclusion:**

This is the first study to investigate the risk of perioperative complications of hepatitis B in Chinese TJAs patients. In consideration of the large population of HBV infection in China, more attention and medical care should be provided to patients with HBV infection who need to undergo TJA operation.

## Background

Chronic hepatitis B virus (HBV) infection remains a major global public health problem, although effective vaccines and antiviral therapy have been carried out [1]. HBV infection is a major cause of liver cancer and cirrhosis of the liver [2], it is one of the most prevalent infectious diseases in mainland China. More than 130 million people are chronically HBV infected, comprising a third of HBV carriers worldwide [3]. The demand for surgical treatment of degenerative diseases, including joint replacements, is expected to grow in aged patients with HBV [4].

Total joint arthroplasty (TJAs) increases in a dramatic speed as the Chinese population ages [5]. The total knee replacement and total hip arthroplasty increase rapidly in China and the number of the operation is over 40,000 per year [6]. With the rising number of TJAs, the number of complications during TJA operation also increases. It has been recognized that there was a need to improve the identification of risk factors and comorbidities associated with complications in order to prevent the social and financial burdens in TJA patients.

Studies revealed an increased hospital stay, mortality, readmission, reoperation and expenses in TJA patients with cirrhosis than those without cirrhosis [5, 7–9]. Furthermore, hepatitis C infection is an increasingly common comorbidity among patients receiving TJAs [4]. However, very few studies showed the effect of HBV infection on the complications of joint arthroplasty [10]. In this study we employed the hospital follow-up database to address this problem and to investigate the hypothesis that HBV infection increases the risk of adverse outcomes in TJA perioperation. Specifically, we can also evaluate basic characteristics of the HBV infection among the patients undergoing TJAs.

## Methods

### Data source

Huashan Hospital follow-up system (HSFS) is a database established for the inpatient and outpatient from 2013. The HSFS comprises complete medical records. HSFS adopts International Classification of Diseases, 9th Revision, Clinical Modification (ICD-9-CM). All data are anonymously analyzed. The informed consent was written by each of the patients and their guardians. The institutional review boards (IRB) at Fudan University Huashan Hospital approved this study protocol.

### Patients

The TJAs cohort was established and patients undergone primary total knee arthroplasty (TKAs) and total hip arthroplasty (THAs) following ICD-9-CM codes were recruited from 2013 to 2016 in Huashan Hospital (Shanghai, China). All individuals who were HBV infected before the TJAs were identified using the ICD-9-CM codes. Furthermore, we excluded patients with human immunodeficiency virus (ICD-9-CM 042) or hepatitis A, C, D, or E virus infection (ICD-9-CM 070.x) to reduce the confounding of HBV infection. Patients with missing data for one or more matching variables were not included. For the safety of surgery, we recommended that all patients with acute recovery HBV should undergo anti-viral therapy before surgery. Therefore, the patients recruited were all virus-carrying or stable HBV after treatment.

We established a non-B hepatitis cohort to match the case cohort with the aim of reducing the effect the demographic characteristics on the study results. We divided patients into two cohorts, as follows: 1) a case cohort of patients with hepatitis B who underwent TJAs, and 2) a non-hepatitis B cohort matching the case cohort adapted method [4] to identify three controls for each patient with hepatitis B. General anesthesia was used in all the patients. Demographic characteristic data were collected from the medical system designed by Microsoft Corporation, including age, gender, and prevalence of comorbidities. By chart review and telephone, these variables were confirmed again for the details. Particularly, regarding a few variables involving postoperative complications that may not be obtained from the system, the regular telephone follow-ups in the outpatients were performed as supplements. We also employed the Deyo scoring, which was a clinical comorbidity index used with ICD-9-CM to evaluate comorbidity variables [11]. The 17 comorbidities (with the point value in parentheses) are as follows:

- congestive heart failure (1 point)
- peripheral vascular disease (1 point)
- dementia (1 point)
- cerebrovascular disease (1 point)
- chronic pulmonary disease (1 point)
- rheumatologic disease (1point)
- peptic ulcer disease (1 point)
- mild liver disease (1 point)
- past myocardial infarct (1 point)
- uncomplicated diabetes (1 point)
- hemiplegia or paraplegia (2 points)
- renal disease (2 points)
- malignancy including leukemia and lymphoma (2 points)
- diabetes with end-stage organ damage (2 points)
- moderate or severe liver disease (3 points)
- metastatic solid tumor (6 points)
- human immunodeficiency virus (HIV) infection (6 points)

Patients whose comorbidities were absent received a score of zero points. We divided patients into three groups, as follows: Deyo score categories of 0, 1, and 2 points.

The matched cohort was chosen by using the matching criteria, as follows: year of surgery, surgical procedure, age, sex, surgically treated joint and surgeon. Each matching cohort (one patient with hepatitis B and three matched controls) was obtained based on a stratification number in the conditional regression modeling. Laboratory tests, including hepatitis biomarkers, hemoglobin (Hb), erythrocyte sedimentation rate (ESR), white blood count (WBC), platelet (PLT), aspartate aminotransferase (AST), alanine aminotransferase (ALT), and C-reactive protein (CRP) were extracted as variables. Aspartate aminotransferase to platelet ratio index (APRI) is a tool applied to evaluate the stage of cirrhosis. The formula of APRI shows as following:

$$ \mathrm{APRI}=\mathrm{AST}\ \mathrm{level}\ \left(\mathrm{upper}\ \mathrm{limit}\ \mathrm{of}\ \mathrm{normal}\right)/\mathrm{platelet}\ \mathrm{counts}\ {\left({10}^9/\mathrm{L}\right)}^{\ast }\ 100 $$

We collected the medical and surgical complications as outcome values. Medical complications include death during admission, acute myocardial infarction and many other major common complications. The detailed information was shown in the Table 2. Surgical complications include wound complications, periprosthetic infection, irrigation and debridement, and postoperative dislocation [4, 12]. All the 196 patients were evaluated clinically and radiologically.

### Statistical analysis

Pearson Chi-square analysis was used to analyze the demographics of the full, unmatched cohort and the postoperative complications. All matched analyses were unadjusted for covariate, as matching for these variables already had minimized their associated confounding effects. Risk was defined as the 95% confidence interval (CI) of a complication for the two cohorts. All statistical analyses were performed using IBM SPSS Statistics (Version 19 New York, USA). For all the analysis, *P* < 0.05 was considered statistical significant.

## Results

A total of 879 patients who underwent primary THA and TKA who met the inclusion and exclusion criteria were included in the study. Among all the patients, 338 (38.5%) of these patients underwent TKA and 571 (61.5%) of these patients underwent THA. The hepatitis B cohort was younger (55.07 ± 5.12 years compared with non-hepatitis B cohort (66.81 ± 6.37 years; *P* < 0.01), with more male patients (53.1% compared with 36.0%; *p* < 0.01), and has a Deyo score of one (33% compared with 25%; *p* < 0.01) or two points (16% compared with 7%; *p* < 0.01) (Table 1).

**Table 1:** Demographic characteristics of patients undergoing TJA

Parameter	HBV	Non-hepatitis B	*P* value
Number	49	830	
Age [mean (SD)] Year	55.07 ± 5.12	66.81 ± 6.37 year	*P* < 0.01
BMI [mean (SD)] Kg/M^2	25.48 ± 2.4	25.89 ± 3.11	0.3643
Sex			*P* < 0.01
Male	26	290	0.0024
Female	23	540	
Surgery			0.0007
THA	19	522	
TKA	30	308	
Deyo score ^a^(points)			*P* < 0.01
0	51%	68%	
1	33%	25%	
≥ 2	16%	7%	

^a^The values are provided as the percentage of patients

Among the non-hepatitis patients, we selected 147 matched control subjects as the HBV(−) group. The total complication rate in patients with hepatitis B (10.2% compared to 4.7%, *P* < 0.01) was higher than in HBV(−) group, including surgery-related complications (6.1% compared to 3.4%), general medical complications (4.1% compared to 1.3%) (Table 2). Applied with regression models, patients with hepatitis B infection had a 25% increased risk (95% CI, 1.04–1.46; *p* < 0.01) of total complication compared with HBV(−) group, as well as a similar result regarding medical and surgical complications. HBV carriers had 31% more risk (95% CI, 1.02–1.62; *p* < 0.01) for medical complication and 18% more risk (95% CI, 1.10–1.26; p < 0.01) for surgical complication.

**Table 2:** Perioperative complications rate and risk for the match cohort

	HBV (+)	HBV (−)	*P*-value	Odds Ratio (95% CI)
Total complications^a^	10.2	4.7	*p* < 0.01	1.25 (1.04–1.46)
Surgery related complications	6.1	3.4	*p* < 0.01	1.31 (1.02–1.62)
wound hemorrhage	4	1.3	*p* < 0.01	
wound disruption	0	0.6	*p* < 0.01	
wound infection	2	0.6	*p* < 0.01	
General medical complications	4.1	1.3	*p* < 0.01	1.18 (1.10–1.26)
pneumonia	0	0.6	–	
deep vein thrombosis	2	0.6	*p* < 0.01	
urinary complication	2	0.6	*p* < 0.01	

^a^The values are provided as the percentage of patients

Complication rates in patients with HBV infection were compared with matched non-infected subjects in TKA and THA (Table 3). When stratified by gender, the incidence of complications in HBV-infected women was 8.6%, while for women without HBV the complication rate was 7.2%. Similar result was shown in the male that 11.5% of male HBV cohorts and 3.8% of males without HBV were present to have complications. Regardless of the sex, patients with HBV were associated with a higher risk of perioperative complications and length of stay compared with non-HBV infection patients, as were shown in Table 4.

**Table 3:** Perioperative complications rate and risk for the match cohort in TKA and THA

	TKA			THA		
	HBV (+)	HBV (−)	*P*-value	HBV (+)	HBV (−)	*P*-value
Total complications^a^	10%	4.4%	*p* < 0.01	10%	5.2%	*p* < 0.01
Surgery-related complications	6.7%	3.3%	p < 0.01	5%	1.7%	*p* < 0.01
General medical complications	3.3%	1.1%	p < 0.01	5%	3.5%	*p* < 0.01

^a^The values are provided as the percentage of patients

**Table 4:** Incidence of complications in different genders

	Female			Male		
	HBV (+)	HBV (−)	*P*-value	HBV (+)	HBV (−)	*P*-value
Total complications^a^	8.6	7.2	*p* < 0.01	11.5	3.8	*p* < 0.01
Surgery- related complications	4.3	2.8	*p* < 0.01	7.6	1.9	*p* < 0.01
General medical complications	4.3	4.4	*p* < 0.01	3.8	1.9	*p* < 0.01

^a^The values are provided as the percentage of patients

Between the two groups, preoperative data of average hemoglobin, average WBC, and average erythrocyte sedimentation rate did not show statistical differences. AST and ALT were higher in the patients with HBV infection (*P* = 0.12). However, the average C-reactive protein in the hepatitis B virus infection group was significantly higher than the non-carriers (*P* < 0.01). Furthermore, the average change level of CRP, Hb, and PLT before and after operation was apparently different, and patients with HBV infection had more considerable change (Table 5).

**Table 5:** Perioperative and Postoperative Data for Hepatitis B and Control Groups

	HBV (+)			HBV (−)			*P*-value
	Preoperative	Postoperative	△Change^a^	Preoperative	Postoperative	△Change^a^	
Hb^b^ g/L	127	105	22	125	109	16	0.23
WBC 10^9/L	6.34	15.43	9.09	7.54	14.32	6.78	0.56
PLT 10^9/L	188	166	22	196	173	23	0.34
ESR mm/h	10	25	15	8	23	15	0.25
CRP mg/dL	4	34	30	5	26	21	*P* < 0.01
AST U/L	45	47	2	23	30	7	0.06
ALT U/L	55	65	10	26	27	1	0.04
0–1	25			119			
1–2	15			20			
> 2	9			8			

^a^Change = Preoperative- Postoperative

^b^*Hb* hemoglobin, *WBC* white blood count, *ESR* erythrocyte sedimentation rate, *PLT* platelet, *AST* aspartate aminotransferase, *ALT* alanine aminotransferase, *CRP* C-reactive protein, *APRI* Aspartate aminotransferase to platelet ratio index

When stratified by APRI, the total complications rate was significantly higher in the APRI score of two or greater than that in the APRI score of zero to one (29.4% versus 1.4%, *P* < 0.01). The group of APRI score of 1–2 showed no significant difference when compared with the other two groups, as shown in Table 6.

**Table 6:** Correlation of APRI score with complication rates

APRI	0–1	1–2	> 2	Odds Ratio (95% CI)
Total number	144	35	17	–
Total complications	2	5	5	2.23 (1.47–3.02)
P_1_–_2_ value*	P_1_–_2_ = 0.32			–
P_2_–_3_value**		P_2-3_ = 0.211		–
P_1_–_3_value***	P_1_–_3_ < 0.05			–

*P_1–2_ referred to the comparison between the group of APRI score of 0–1 and 1–2, **P_2–3_ referred to the comparison between the group of APRI score of 1–2 and > 2, and ***P_1–3_ referred to the comparison between the group of APRI score of 0–1 and > 2

## Discussion

China has a considerable number of HBV infection cases, approximately accounting for 8.76% of the population [13]. Moreover, China is simultaneously a country in great need of TJAs. With the improvement of treatment and prevention on HBV, the prevalence of HBV infection in China has appreciably decreased in recent years. However, the cumulative number of patients with hepatitis B is still enormous. Nevertheless, few studies have assessed the impact of HBV infection on the clinical outcomes after total joint arthroplasty operation. Our study aims to address and evaluate basic characteristics of the HBV infection in TJA patients, as well as the difference regarding outcome after TJAs. In our study, a high HBV infection rate is shown among TJAs, and HBV infection is associated with high complications and CRP level. HBV infection is an independent risk factor for complications of the arthroplasty operation.

In our literature review, only one study evaluated the effect of HBV infection on periprosthetic joint infections (PJI) after TKAs among 4196 patients. The author reported that HBV infection was regarded as a risk factor for PJI after TKAs among men; however, it was not a risk factor among the females. In their study, among 124 patients with HBV infection, 2.96% of all the patients underwent TKAs. In our study, we reported a significantly high HBV infection rate of 5.5% of all the TJA patients. This is the first report regarding the rate of HBV infected TJA patients in mainland China which was not only classified by the ICD-9-CM but also confirmed by the lab test. Although the population is small, considering the potential of the outcome after TJAs, more attention should be provided to the special group.

HBV infection appeared to be a more important risk factor for complications in TJA and was independent from other factors. Kuo [10] showed that the risk of PJI in HBV-infected males increased by 4.32-fold compared with men without HBV. We confirmed the significant effect of HBV infection, indicating that HBV might increase the rate of complications after TJAs. Sex and surgical methods did not change the effect of HBV in the subgroup by stratification analysis. Furthermore, patients with HBV infection showed a 25% increased risk of total complication, 31% increased risk of surgery-related complications, and 18% increased risk of general medical complications compared with the match non-infected group using regression models. However these findings were not in agreement with the Jiang et al.’s result [5], in which HBV was found to be not independently associated with periprosthetic joint infections. In the subgroup analysis, we did not find any difference between the surgical methods as well as sex. However, we found significant difference in CRP level between HBV and matched non-infected group. A previous study has reported that persistent CRP levels ≥29 mg/L predicted bad outcomes independently of age, MELD, and co-morbidities [14–17]. In other aspects, HBV infection changes the immune functions, especially when the liver suffers from fibrosis [18]. C-reactive protein is considered associated with HBV replication, liver related damage, and fibrosis, and serum CRP may be a marker for the diagnosis of liver fibrosis in patients with HBV [18, 19]. Furthermore, Joo [20] reported that HBsAg-positive subjects had a higher incidence of thrombocytopenia than healthy controls (11.2 vs 1.9 per 1000 person-years, respectively). Strong associations between HBsAg positivity and thrombocytopenia were consistently observed across prespecified subgroups. The low platelet counts reduce the coagulation function [21] and therefore the secondary changes after HBV infection may be the reason for the increase in the complication rate in the HBV infection group [17, 20, 21].

Chronic hepatitis B virus infection is still the main cause of cirrhosis in China, with average annual decompensated liver cirrhosis of 3%. Considering that numerous patients had no idea that they had been infected by HBV, we also used the APRI scores to assess the severity of liver fibrosis in all patients, aiming to obtain a more accurate liver fibrosis status. High APRI scores predicted a high complication rate in our study (*P* < 0.05), and APRI score two posts a percentage of 29.4% in all the patients indicating that severe live fibrosis leads to high complication rates.

A few limitations exist in our study. First, the sample was small because we collected the information of target patients from 2013 to 2016. However, our study is the first to evaluate the impact of HBV infection on the outcome of TJAs, providing an initial description of complications in TJAs caused by HBV. Second, we could not match all the controlled variables; however, we selected the matched group by a random matching criterion to reduce the selected bias. Third, the clinical history collection system is not perfect to include all the information; hence, a few diagnoses may be underreported. In addition, the dataset used for this study was restricted to medical and diagnoses recorded during the time of hospital stay, which could not provide complete long-term outcomes. In addition, we obtained other outcomes by following-up in the outpatient or by telephone. Another limitation in our cohort is that we could not distinguish if the patients were experiencing acute or chronic HBV infection. However, we confirmed the HBV infection status by clinical laboratory values, providing a more accurate diagnosis than the previously reported study which depended on the code system [22].

## Conclusions

This is the first analysis of the risk of postoperative complications of primary TJAs in Chinese patients with hepatitis B virus infection. Our study shows an increased risk of multiple perioperative complications. In consideration of the large population of HBV infection in China, more attention should be provided to patients with HBV infection who need to undergo TJAs. We suggest a national study to identify the relationship between HBV infection and TJAs to further provide substantial medical analysis to prevent the complications.

## Abbreviations

ALT: Alanine aminotransferase
APRI: Aspartate aminotransferase to platelet ratio index
AST: Aspartate aminotransferase
CI: Confidence interval
CRP: C-Reactive protein
ESR: Erythrocyte sedimentation rate
Hb: Hemoglobin
HBV: Hepatitis B virus
HIV: Human immunodeficiency virus
HSFS: Huashan Hospital follow-up system
ICD-9-CM: International Classification of Diseases, 9th Revision, Clinical Modification
IRB: institutional review boards
PJI: Periprosthetic joint infections
PLT: Platelet
THAs: Total hip arthroplasty
TJA: Total joint arthroplasty
TKAs: Total knee arthroplasty
WBC: White blood count

## References

[1] Schweitzer Aparna, Horn Johannes, Mikolajczyk Rafael T, Krause Gérard, Ott Jördis J (2015). Estimations of worldwide prevalence of chronic hepatitis B virus infection: a systematic review of data published between 1965 and 2013. The Lancet.

[2] He J, Gu D, Wu X, Reynolds K, Duan X, Yao C, Wang J, Chen CS, Chen J, Wildman RP (2005). Major causes of death among men and women in China. N Engl J Med.

[3] Li YY, Chen WW, Wei L, Xie YX, Wang LF, Fu JL, Wang FS (2017). A survey of knowledge about hepatitis B among new military recruits in China. Mil Med Res.

[4] Issa K, Boylan MR, Naziri Q, Perfetti DC, Maheshwari AV, Mont MA (2015). The impact of hepatitis C on short-term outcomes of Total joint arthroplasty. J Bone Joint Surg Am.

[5] Jiang SL, Schairer WW, Bozic KJ (2014). Increased rates of periprosthetic joint infection in patients with cirrhosis undergoing total joint arthroplasty. Clin Orthop Relat Res.

[6] Kunzheng W. Brief discussion on present status and future of joint replacement in China. Chin J Joint Surg (Electronic Version). 2015:703–6.

[7] Hsieh PH, Chen LH, Lee MS, Chen CH, Yang WE, Shih CH (2003). Hip arthroplasty in patients with cirrhosis of the liver. J Bone Joint Surg Br.

[8] Moon YW, Kim YS, Kwon SY, Kim SY, Lim SJ, Park YS (2007). Perioperative risk of hip arthroplasty in patients with cirrhotic liver disease. J Korean Med Sci.

[9] Shih LY, Cheng CY, Chang CH, Hsu KY, Hsu RW, Shih HN: Total knee arthroplasty in patients with liver cirrhosis. J Bone Joint Surg Am 2004, 86-a:335–341. 10.2106/00004623-200402000-00017 14960679

[10] Kuo SJ, Huang PH, Chang CC, Kuo FC, Wu CT, Hsu HC, Lin CC. Hepatitis B virus infection is a risk factor for Periprosthetic joint infection among males after Total knee arthroplasty: a Taiwanese Nationwide population-based study. Medicine (Baltimore). 2016;95. 10.1097/MD.0000000000003806 PMC4900725 27258517

[11] Deyo RA, Cherkin DC, Ciol MA (1992). Adapting a clinical comorbidity index for use with ICD-9-CM administrative databases. J Clin Epidemiol.

[12] Best MJ, Buller LT, Klika AK, Barsoum WK (2015). Increase in perioperative complications following primary total hip and knee arthroplasty in patients with hepatitis C without cirrhosis. J Arthroplast.

[13] Zeng F, Guo P, Huang Y, Xin W, Du Z, Zhu S, Deng Y, Zhang D, Hao Y (2016). Epidemiology of hepatitis B virus infection: results from a community-based study of 0.15 million residents in South China. Sci Rep.

[14] Cervoni JP, Thevenot T, Weil D, Muel E, Barbot O, Sheppard F, Monnet E, Di Martino V (2012). C-reactive protein predicts short-term mortality in patients with cirrhosis. J Hepatol.

[15] Di Martino V, Coutris C, Cervoni JP, Dritsas S, Weil D, Richou C, Vanlemmens C, Thevenot T (2015). Prognostic value of C-reactive protein levels in patients with cirrhosis. Liver Transpl.

[16] Dupont C, Rodenbach J, Flachaire E (2008). The value of C-reactive protein for postoperative monitoring of lower limb arthroplasty. Ann Readapt Med Phys.

[17] Huang SS, Xie DM, Cai YJ, Wu JM, Chen RC, Wang XD, Song M, Zheng MH, Wang YQ, Lin Z, Shi KQ (2017). C-reactive protein-to-albumin ratio is a predictor of hepatitis B virus related decompensated cirrhosis: time-dependent receiver operating characteristics and decision curve analysis. Eur J Gastroenterol Hepatol.

[18] Ma LN, Liu XY, Luo X, Hu YC, Liu SW, Tang YY, Pan JL, Ding XC (2015). Serum high-sensitivity C-reactive protein are associated with HBV replication, liver damage and fibrosis in patients with chronic hepatitis B. Hepatogastroenterology.

[19] Zhu S, Waili Y, Qi X, Chen Y, Lou Y, Chen B (2017). Serum C-reactive protein predicts early mortality in hospitalized patients with HBV-related decompensated cirrhosis. Medicine (Baltimore).

[20] Joo EJ, Chang Y, Yeom JS, Lee YG, Ryu S (2017). Hepatitis B infection is associated with an increased incidence of thrombocytopenia in healthy adults without cirrhosis. J Viral Hepat.

[21] Hafeez M, Sarfraz T, Khan RG, Rafe A, Rasool G, Ahmed KN (2016). Hepatitis B leading to megaloblastic Anemia and catastrophic peripheral thrombocytopenia. J Coll Physicians Surg Pak.

[22] Tiberi JV, Hansen V, El-Abbadi N, Bedair H (2014). Increased complication rates after hip and knee arthroplasty in patients with cirrhosis of the liver. Clin Orthop Relat Res.

